# Psychometric properties of the Chinese version of the Hypertension Belief Assessment Tool

**DOI:** 10.1186/s12877-024-04853-1

**Published:** 2024-04-25

**Authors:** Xue Yang, Yujin Mei, Yuqing Li, Xiaoyun Zhang, Jiaofeng Gui, Ying Wang, Wenyue Chen, Mingjia Chen, Changjun Liu, Lin Zhang

**Affiliations:** 1 School of Nursing, Wannan Medical College, 22 Wenchang West Road, Higher Education Park, 241002 Wuhu City, An Hui Province P.R. China; 2 School of Marxism, Jinzhou Medical University, No. 40, Section 3, Songpo Road, Linghe District, 121001 Jinzhou City, Liaoning Province P.R. China; 3 Department of Internal Medicine Nursing, School of Nursing, Wannan Medical College, 22 Wenchang West Road, Higher Education Park, 241002 Wuhu City, An Hui Province P.R. China

**Keywords:** Reliability, Validity, HBAT, Beliefs, Hypertensive patients

## Abstract

**Background:**

Hypertension is prevalent in China. Hypertensive patients suffer from many health problems in life. Hypertension is a common chronic disease with long-term and lifelong characteristics. In the long run, the existence of chronic diseases will affect the patient’s own health beliefs. However, people’s health beliefs about Hypertension are not explicit. Therefore, it is vital to find a suitable instrument to comprehend and improve the health beliefs of hypertensive patients, thus, better control of blood pressure and improvement of patient’s quality of life are now crucial issues. This study aimed to translate the Hypertension Belief Assessment Tool (HBAT) into Chinese and examine the psychometric properties of the Chinese version of the Hypertension Belief Assessment Tool in hypertensive patients.

**Methods:**

This is a cross-sectional study. We translated the HBAT into Chinese and tested the reliability and validity of the Chinese version among 325 hypertensive patients.

**Results:**

The Chinese version of the scale contains 21 items. The Exploratory Factor Analysis (EFA) revealed six factors and explained 77.898% of the total variation. A six-factor model eventually showed acceptable fit indices in the Confirmatory Factor Analysis (CFA). With modified Confirmatory Factor Analysis, the fit indices were Chi-square/Degree of Freedom (CMIN/DF) = 2.491, Comparative Fit Index (CFI) = 0.952, Incremental Fit Index (IFI) = 0.952, Root-mean-square Error of Approximation (RMSEA) = 0.068, Tucker Lewis Index (TLI) = 0.941. The HBAT exhibits high internal consistency reliability (0.803), and the scale has good discriminant validity.

**Conclusion:**

The results suggest that the HBAT is a reliable and valid instrument for assessing the beliefs of Chinese hypertensive patients.

## Introduction

With the development of technology and science, the prevalence of Hypertension is also increasing rapidly. Hypertension, one of the major chronic diseases [1], is a common cause and risk factor for peripheral arterial, cardiovascular, cerebrovascular disease, and renal failure [2, 3]. Hypertension is a global disease. Many findings show that Hypertension has become a major public health problem, such as Western Africa [4], the Middle East [5], Mayotte [6], Mexico [7], and Tehran [8]. Globally, 49% (46–52) of men and 59% (55–62) of women reported a diagnosis of Hypertension in 2019, and 38% (35–41) of men and 47% (43–51) of women were treated. Control rates among people with Hypertension were 18% (16–21) for men and 23% (20–27) for women [9]. In China, the situation of Hypertension is poor [10, 11]. The prevalence, awareness, treatment, and control of Hypertension among hypertensive patients ranged from 18.0 to 44.7%, 23.6–56.2%, 14.2–48.5%, and 4.2–30.1% respectively [12].

Studies have pointed out that beliefs about chronic disease were related to chronic disease behaviors [13]. As we all know, belief is a psychological term. Hypertension beliefs reflect a range of knowledge, perceptions, and behaviors about Hypertension. The beliefs about Hypertension have an association with the treatment of Hypertension, and the beliefs also affect patients’ adherence [14]. Based on the Health Belief Model (HBM), there were plenty of vital elements affecting Hypertension actions among patients such as self-efficacy, benefits, barriers, susceptibility, and severity [15, 16]. The HBM claims that one’s health habits could affect their beliefs and attitudes [17]. HBM, which contains perceived self-efficacy, will provide a more robust approach to comprehending and influencing health-related behavior [18, 19]. A randomized controlled trial found that positive health beliefs can promote blood pressure reduction [20]. A cross-sectional study also found that patients’ beliefs are important to improve medication adherence [21]. Clinically, patients’ beliefs should be assessed, and strategies to improve medication adherence beliefs have potent influences on actions and lifestyles [22, 23]. Greater awareness and perceptions of illness of the health beliefs of older adults with Hypertension can help them to manage their blood pressure, including self-management [24, 25]. Successful self-management of chronic diseases depends on behavior. People with high self-efficacy, and better caregiver contribution are more likely to maintain healthy behaviors and self-care for chronic illness [26, 27]. Therefore, beliefs about Hypertension and its complications are crucial to Hypertension management behaviors [28].

However, there are few studies on health beliefs about Hypertension in China. At present, there is a large number of hypertensive people in China, and there is no relevant tool for assessing the health beliefs of hypertensive patients in China. The situation of health beliefs of people with Hypertension is unclear. Therefore, we aim to translate the appropriate English scale into Chinese. We hope that through our study, we can understand the current status of the health beliefs of Chinese hypertensive patients and help Chinese hypertensive patients improve their health beliefs, to effectively improve the rate of Hypertension control and awareness of prevention. It is instrumental to seek a sensible tool to measure people’s beliefs, motivation, high blood pressure-health literacy, self-efficacy, and behavior [29]. The Hypertension self-care questionnaire included in medication therapy, follow-up, promoting qualifications, healthy lifestyle, and following recommendations [30]. All dimensions are highly relevant to the management of hypertensive patients. However, these 5 dimensions are primarily used to measure self-care in hypertensive patients and do not apply to the assessment of health beliefs. The Knowledge-Level Scale (Six dimensions: definition, drug compliance, medical treatment, diet, complications, and lifestyle) was conducted to assess knowledge about Hypertension [31]. This scale will be important in measuring the knowledge level of patients with Hypertension. The Chronic Disease Self-Efficacy Scale was a five-factor scale [32], However, the scale has limitations in measuring beliefs about Hypertension. Based on the HBM, many scholars have developed scales suitable for various diseases. The results indicated that the instrument was reliable and valid [33]. There are several tools for measuring health beliefs, such as The Beliefs About Medicines Questionnaire [34] and The Health Belief Model Questionnaire [24]. However, these scales focus only on patients’ perceptions of medications and medication adherence and do not provide a comprehensive measure of patients’ hypertension health beliefs. Other scales currently used to measure health beliefs are the Health Belief Model Scale and the Mental Illness Beliefs Scale. However, the Health Belief Model Scale can only be used in the assessment of testicular cancer in men to measure susceptibility, severity, health motivation, barriers, benefits, and self-efficacy [17]. The Mental Illness Beliefs Scale is only used to assess beliefs about mental illness [35].

Health beliefs have many components. For older patients with Hypertension, the Hypertension Beliefs Assessment Tool was the first to explore an individual’s belief towards Hypertension based on the HBM [36]. Therefore, our study will test the validity and reliability of the HBAT, which will be helpful to enhance the patient’s health beliefs, and knowledge of the disease and improve the patients’ quality of life by this study. Overall, this study aims to translate the English version of HBAT into Chinese and test the validity and reliability of HBAT in hypertensive patients. And provide more suggestions for the prevention and intervention of chronic diseases.

## Methods

### Design and sample

A cross-sectional survey was conducted from June to November 2022 in the Anhui Province, China. 325 hypertension patients in the First Affiliated Hospital of Wannan Medical College participated in this questionnaire. We obtain approval from the College of Nursing’s Research Committee at Wannan Medical College for the study.

The inclusion criteria: older than 45 years; able to communicate and listen; meet the diagnostic criteria of Hypertension [37]; taking medication for at least one year; consent to participate. Exclusion criteria: patients with mental disorders and secondary hypertension; uncooperative participants.

### The instrument

The 23-item, HBAT was first used in Northwest Ethiopia, and developed by Teshome [36]. The HBAT incorporates six dimensions: Perceived susceptibility to hypertension (item1-4), Self-efficacy (item21-23), Perceived socio-economic-related severity (item10-13), Perceived benefits of taking action (item14-16), Perceived barriers to taking action (item17-20) and Perceived severity of hypertension (item5-9). The HBAT has a 5-point Likert scale, with the answer choices ranging from “very disagree” to “very agree.” A high score on the scale indicates a strong sense of belief. In the original scale, Cronbach’s alpha was 0.85 for the entire scale, ranging from 0.74 to 0.92 for the sub-domains. The content validity index of the overall scale (0.96), and kappa coefficient of agreement ranged from 0.8 to 1. The Average Variance Extracted were above the cut-off value of 0.5 for all factors, ranging from 0.55 to 0.82 [36].

### Translation procedure

Translation steps conformed to the Brislin’s translation model [38]. Two bilingual professional translators translated the HBAT from English into Chinese at first (Version A1 and Version A2). Then another two translators who were proficient in English and Medicine translated the Chinese version (Version A1 and Version A2) back into English (Version B1 and Version B2). Second, two nursing experts and one psychology expert compared and discussed Version A1 and A2, Version B1 and B2, respectively, and came up with translated Version A and back-translated Version B. The original scale, translated Version A, and back-translated Version B were sent to the above participants. Experts were asked to compare and discuss the original scale and the back-translated Version B and then evaluated the language habits of the whole scale [39, 40]. After the third round of discussions, the translated Version A was adjusted to form the initial Version C of the scale. Third, we carry out a preliminary experiment on 30 hypertensive patients. After the pre-survey, the patient’s understanding of the questionnaire was good. Finally, the Chinese Version D of the scale was formed by synthesizing the feedback from the pre-survey and the experts’ suggestions. During the translation of the scale, the consensus of the translators was accomplished by following the Delphi method [41]. The English and Chinese versions of HBAT are shown in Table 1.

**Table 1:** The Hypertension Belief Assessment Tool Scale (English version and Chinese version)

Items	Item content (English/Chinese)	Scores
Q1	My chance of developing hypertension is high	1 2 3 4 5
	我患高血压的几率很高	
Q2	The possibility that I will develop hypertension in a few years is very high	1 2 3 4 5
	几年后, 我患高血压的可能性非常大	
Q3	I can have hypertension even without the feeling of its symptoms	1 2 3 4 5
	即使没有症状, 我也会患上高血压	
Q4	I am more likely to catch HTN than other people	1 2 3 4 5
	我比其他人更容易患高血压	
Q5	Hypertension is a serious disease	1 2 3 4 5
	高血压是严重的疾病	
Q6	Hypertension is a lifelong disease	1 2 3 4 5
	高血压是终身的疾病	
Q7	Complication of hypertension can cause permanent damage	1 2 3 4 5
	高血压的并发症可造成永久性损害	
Q8	Hypertension can cause death	1 2 3 4 5
	高血压能导致死亡	
Q9	Sudden falling may happen due to hypertension	1 2 3 4 5
	高血压可能导致突然跌倒	
Q10	Hypertension will cause dependence on others 高血压会导致依赖他人	1 2 3 4 5
Q11	Hypertension can threaten patients’ relationship with their family	1 2 3 4 5
	高血压会严重影响患者与家人间的关系	
Q12	Hypertension can cause sexual dysfunction 高血压会导致性功能障碍	1 2 3 4 5
Q13	Hypertension can cause financial burden	1 2 3 4 5
	高血压会增加经济负担	
Q14	Early detection of HTN makes prevention of complications easier	1 2 3 4 5
	早期发现高血压更容易预防并发症	
Q15	Timely initiation of treatment makes prevention of complications easier	1 2 3 4 5
	及时开始治疗可以更容易地预防并发症	
Q16	Keeping blood pressure close to normal prevents hypertension complications	1 2 3 4 5
	保持血压接近正常可预防高血压并发症	
Q17	Lack of health information can prevent getting healthcare services	1 2 3 4 5
	由于缺乏健康信息, 我很难获得医疗服务	
Q18	Lack of transportation makes it difficult for me to get healthcare service	1 2 3 4 5
	交通不便使我很难获得医疗服务	
Q19	The cost of screening service makes it difficult for me to get the service	1 2 3 4 5
	检查服务的费用使我很难获得医疗服务	
Q20	Lack of nearby health facilities makes it difficult for me to get services 附近缺乏医疗机构使我去医院看病变得困难	1 2 3 4 5
Q21	I can know my status by checking my BP regularly	1 2 3 4 5
	通过规律监测血压我可以了解自身状况	
Q22	I am sure of when to contact health workers while I am feeling unusual health conditions	1 2 3 4 5
	当我感觉到异常的健康状况时, 我知道何时联系医生	
Q23	I follow health information about hypertension	1 2 3 4 5
	我遵从有关高血压的健康信息	

### Data collection

Data were collected between June and November 2022 through face-to-face interviews with hypertensive patients. The investigator will complete the questionnaire based on the patient’s responses. Convenience sampling methods were used in our study. We selected hypertensive patients from the Department of Cardiovascular Medicine and the Department of Geriatrics of the First Affiliated Hospital of Wannan Medical College as our study participants. We also collected sociodemographic information such as the sex and age of the patients, and the HBAT scale was mainly used to collect information on the health beliefs of hypertension patients. A total of two full-time postgraduate nursing students participated in the survey. All the investigators have completed a standardized training program (The object of the questionnaire, The way of asking questions, Points to note when filling out the questionnaire, The quality control of the questionnaire). In the course of the investigation, each questionnaire will takes about 20 mins. All participants expressed their willingness to cooperate with the investigation.

Blood pressure measurements were taken by one person. Blood pressure was measured early in the morning, when the patient was quiet and not taking any antihypertensive medication, with the brachial artery of the upper limb as the main measuring position, using an Omron sphygmomanometer (Version U701)) to measure the blood pressure, when measuring the blood pressure, make sure that the patient’s upper limb was kept at the same level with the heart and the sphygmomanometer, a total of three measurements were taken at intervals of five minutes, and finally take the average value of the three measurements as the patient’s final blood pressure. And according to the grading diagnostic criteria for Hypertension [42], we divided blood pressure level into three levels: under 140/90mmHg, 140–160/90-100mmHg, and above 160/100mmHg. In the end, 325 hypertensive patients participated in the survey.

### Statistical analysis

Statistical analysis was conducted with the IBM SPSS Statistics 22 software package. Scale and factor analyses were conducted to verify the reliability and validity of the questionnaire. We used descriptive analyses to describe the demographic information. The reliability of the HBAT was calculated by Item-total score correlations, Cronbach’s alpha, and retest reliability. Validity tests were analyzed by content validity and construct validity. The purpose of EFA is to assess the factor structure, where each factor loading reflects the contribution of the scale question item to that dimension. Higher factor loadings indicate that the item is more closely related to the dimension. EFA was used to measure the construct validity of the HBAT. In EFA, the Kaiser–Meyer–Olkin (KMO) should be greater than 0.70, and the principal component analysis was used to extract at least four factors with Eigenvalues greater than 1. The contribution of the total variance should be higher than 60% [43]. CFA is to determine the number of factors and the correspondence between each item and factor. CFA was performed using AMOS 21.0 software to assess the structural model fit of HBAT. In CFA, standardized factor loadings should be > 0.30, Chi-square/Degrees of Freedom < 3 or RMSEA < 0.08 [44]. To investigate the discriminant validity of the scale, a two-tailed independent samples t-test was used in the study. The analysis results were evaluated within the 95% confidence interval, and the statistical significance limit was accepted as *P* < 0.05 [45].

## Results

### The sample

A total of 325 participants (including 168 males and 157 females) aged 45 to 91 years old completed the baseline questionnaire. The mean age was 70.56 ± 10.57. More details are shown in Table 2.

**Table 2:** Characteristics of study participants (*N* = 325)

Variables	Categories	N	Percentage/$\overline{\boldsymbol X}$±S
**Sex**	Male	168	51.69%
	Female	157	48.31%
**Age (years)**	45–91	325	70.56 ± 10.57
**Marriage**	Single	7	2.15%
	Married	248	76.31%
	Widowed	70	21.54%
**Education**	Illiteracy	89	27.38%
	Primary school	86	26.46%
	Junior high school	66	20.32%
	Senior high school	47	14.46%
	College school	37	11.38%
**Monthly income(CNY)**	< 2000	132	40.61%
	2001–3000	28	8.62%
	3001–4000	64	19.69%
	> 4000	101	31.08%
**Residence**	City	100	30.77%
	Suburb	84	25.85%
	Rural	141	43.38%
**Duration of hypertension**	< 5 years	58	17.85%
	6–10 years	75	23.07%
	11–20 years	96	29.54%
	> 20 years	96	29.54%
**Family history**	Yes	171	52.62%
	No	154	47.38%
**Blood pressure control level**	Under 140/90mmHg	215	66.16%
	140–160/90–100 mmHg	73	22.46%
	Above 160/100 mmHg	37	11.38%
**Medical insurance**	Rural medical insurance	140	43.08%
	Urban medical insurance	182	56.00%
	None	3	0.92%

### Item analysis

We calculate the Pearson correlation coefficient of each item score and the total score of the scale. A corrected Item-total correlation should be above 0.20 [36, 46]. If t < 3.00 or *P* > 0.05, considering deleting it. Mean values for each item ranged from 2.338 to 4.809 (Table 3). The low item was “The possibility that I will develop hypertension in a few years is very high,” indicating that most patients had poor belief in Hypertension. The high item was “Timely initiation of treatment makes prevention of complications easier”, indicating that the majority of the patients noticed the benefits of taking action.

**Table 3:** Item-total score person correlation analysis results in HBAT of 23 items (*N* = 325, α = 0.05)

Items	Item content	Means	* r*	* P*
**Q1**	My chance of developing hypertension is high	2.692	0.644	< 0.001
**Q2**	The possibility that I will develop hypertension in a few years is very high	2.338	0.610	< 0.001
**Q3**	I can have hypertension even without the feeling of its symptoms	2.560	0.674	< 0.001
**Q4**	I am more likely to catch HTN than other people	2.713	0.677	< 0.001
**Q5**	Hypertension is a serious disease	4.073	0.462	< 0.001
**Q6**	Hypertension is a lifelong disease	4.738	0.260	< 0.001
**Q7**	Complication of hypertension can cause permanent damage	4.683	0.349	< 0.001
**Q8**	Hypertension can cause death	3.855	0.290	< 0.001
**Q9**	Sudden falling may happen due to hypertension	2.760	0.325	< 0.001
**Q10**	Hypertension will cause dependence on others	2.418	0.433	< 0.001
**Q11**	Hypertension can threaten patients’ relationship with their family	2.356	0.441	< 0.001
**Q12**	Hypertension can cause sexual dysfunction	2.443	0.426	< 0.001
**Q13**	Hypertension can cause financial burden	4.024	0.350	< 0.001
**Q14**	Early detection of HTN makes prevention of complications easier	4.784	0.376	< 0.001
**Q15**	Timely initiation of treatment makes prevention of complications easier	4.809	0.410	< 0.001
**Q16**	Keeping blood pressure close to normal prevents hypertension complications	4.784	0.375	< 0.001
**Q17**	Lack of health information can prevent getting healthcare services	2.550	0.348	< 0.001
**Q18**	Lack of transportation makes it difficult for me to get healthcare service	2.400	0.437	< 0.001
**Q19**	The cost of screening service makes it difficult for me to get the service	2.480	0.418	< 0.001
**Q20**	Lack of nearby health facilities makes it difficult for me to get services	2.372	0.469	< 0.001
**Q21**	I can know my status by checking my BP regularly	4.452	0.398	< 0.001
**Q22**	I am sure of when to contact health workers while I am feeling unusual health conditions	4.560	0.388	< 0.001
**Q23**	I follow health information about hypertension	3.033	0.252	< 0.001

For the 23-item HBAT, there were outstanding differences in Item-total score correlations, and the coefficient fluctuated between 0.252 and 0.677 (Table 3). The correlation coefficient of Q23 was the lowest on the whole scale. Therefore, Q23 was eventually discarded.

### Content validity

Six nursing experts were invited to evaluate the content validity of the questionnaire, and the results showed that the Item-level Content Validity Index (I-CVI) was 0.83 ~ 1.00, and the Scale-level Content Validity Index (S-CVI) was 0.84, which is acceptable [47]. The process and results of the validation of this study are presented in Table 4.

**Table 4:** Process and results of validation

Process	Results
Content validity	The Item-level Content Validity Index (I-CVI) was 0.83 ~ 1.00, and the Scale-level Content Validity Index (S-CVI) was 0.84
Construct validity	1. Exploratory Factor Analysis (1) The principal component analysis (2) Six factors with an Eigenvalue of > 1 (3) Kaiser–Meyer–Olkin (0.762) and Bartlett spherical test value 5401.941 (*df* = 210, *P* < 0.001) (4) Explained 77.898% of the total variance
	2. Confirmatory Factor Analysis CMIN/DF = 2.491, CFI = 0.952, IFI = 0.952, TLI = 0.941, and RMSEA = 0.068
Discriminant validity	The independent sample t-test The *P* value < 0.001

### Exploratory factor analysis

The KMO values greater than 0.70 indicate suitability for factor analysis [43]. In the 22 items HBAT, the principal component analysis and scree plot also verified six factors with an Eigenvalue of > 1 [36]. The first factor accounted for 20.785% of the total variance. The second factor accounted for 14.791% of the total variance. Factor three, four, five, and six accounted for 14.222%, 12.975%, 7.188%, and 5.299% of the total variance, respectively. After maximum variance rotation, six common factors were extracted, and it explained 73.014% of the total variance (Table 5). The rotated component matrix demonstrated that Q1 to Q4 loading ranging from 0.951 to 0.965 (F1); Q17 to Q20 loading ranging from 0.703 to 0.945 (F2); Q9 to Q12 loading ranging from 0.753 to 0.891 (F3); Q14 to Q16 loading ranging from 0.881 to 0.925 (F4); Q21 to Q22 loading ranging from 0.762 to 0.773 (F5); Q5 to Q8 loading ranging from 0.568 to 0.787 (F6) (Table 5).

**Table 5:** Factor load and communalities of each item in HBAT of 22 items (*N* = 325)

Items	F1	F2	F3	F4	F5	F6	Communalities
**Q1**	**0.963**	0.037	0.017	0.000	0.073	0.019	0.935
**Q2**	**0.869**	0.017	0.130	-0.011	-0.015	0.086	0.780
**Q3**	**0.965**	0.053	0.036	0.027	0.062	0.055	0.943
**Q4**	**0.951**	0.073	0.015	0.046	0.105	0.028	0.924
**Q10**	0.072	0.021	**0.889**	0.046	0.004	0.034	0.800
**Q11**	0.096	0.037	**0.891**	0.027	-0.032	0.046	0.809
**Q17**	0.018	**0.703**	0.154	0.059	-0.220	-0.037	0.571
**Q18**	0.042	**0.945**	-0.032	-0.020	0.026	0.020	0.897
**Q19**	0.053	**0.919**	-0.067	-0.033	0.020	0.025	0.855
**Q20**	0.045	**0.917**	0.052	0.023	0.008	0.030	0.847
**Q12**	0.055	0.060	**0.776**	0.008	0.233	-0.020	0.664
**Q13**	0.026	0.439	0.006	0.087	0.276	0.019	0.278
**Q14**	0.019	0.037	0.029	**0.881**	0.231	0.129	0.849
**Q15**	-0.001	0.056	0.105	**0.925**	0.211	0.137	0.934
**Q16**	0.030	0.023	0.089	**0.900**	0.186	0.069	0.860
**Q21**	0.109	0.179	-0.063	0.160	**0.773**	0.142	0.692
**Q22**	0.087	0.007	-0.008	0.331	**0.762**	0.148	0.720
**Q5**	0.156	0.088	0.294	-0.027	0.118	**0.623**	0.521
**Q6**	-0.022	0.033	-0.118	0.124	0.134	**0.756**	0.621
**Q7**	0.026	0.002	-0.019	0.130	0.230	**0.787**	0.690
**Q8**	0.079	-0.111	0.295	0.153	-0.323	**0.568**	0.556
**Q9**	-0.050	-0.022	**0.753**	0.155	-0.239	0.163	0.677

F1 contained Q1, Q2, Q3, Q4, F2 contained Q17, Q18, Q19 and Q20, F3 contained Q9, Q10, Q11, and Q12, F4 contained Q14, Q15, Q16, F5 contained Q21, and Q22, F6 contained Q5, Q6, Q7and Q8

The loading on Q13 was lower than 0.5, so we removed the Q13. Finally, we keep the 21 items HBAT (Table 6). In EFA of 21 items HBAT, the KMO was 0.762 and the Bartlett spherical test value was 5401.941(*df* = 210, *P* < 0.001). Overall, the six common factors explained 77.898% of the total variance (Table 6). The rotated component matrix demonstrated that Q1 to Q4 strongly loaded on factor 1 with factor loading ranging from 0.867 to 0.966; Q17 to Q20 strongly loaded on factor 2 with factor loading ranging from 0.740 to 0.944; Q9 to Q12 strongly loaded on factor 3 with factor loading ranging from 0.749 to 0.891; Q14 to Q16 strongly loaded on factor 4 with factor loading ranging from 0.897 to 0.938; Q5 to Q8 strongly loaded on factor 5 with factor loading ranging from 0.557 to 0.797; Q21 to Q22 strongly loaded on factor 6 with factor loading ranging from 0.769 to 0.857 (Table 6).

**Table 6:** Factor load and communalities of each item in HBAT of 21 items (*N* = 325)

Items	F1	F2	F3	F4	F5	F6	Communalities
**Q1**	**0.965**	0.034	0.018	0.005	0.020	0.061	0.936
**Q2**	**0.867**	0.015	0.136	-0.018	0.081	0.002	0.777
**Q3**	**0.966**	0.047	0.037	0.031	0.055	0.047	0.943
**Q4**	**0.954**	0.065	0.015	0.056	0.031	0.081	0.925
**Q5**	0.151	0.071	0.312	-0.035	**0.616**	0.167	0.534
**Q6**	-0.016	0.019	-0.119	0.142	**0.764**	0.085	0.625
**Q7**	0.033	-0.014	-0.020	0.155	**0.797**	0.166	0.688
**Q8**	0.075	-0.069	0.294	0.117	**0.557**	-0.350	0.544
**Q9**	-0.052	0.000	**0.749**	0.136	0.155	-0.270	0.678
**Q10**	0.070	0.022	**0.891**	0.047	0.029	-0.006	0.802
**Q11**	0.094	0.043	**0.891**	0.027	0.040	-0.045	0.809
**Q12**	0.060	0.035	**0.773**	0.038	-0.015	0.185	0.639
**Q14**	0.023	0.003	0.032	**0.897**	0.136	0.164	0.852
**Q15**	0.001	0.034	0.108	**0.938**	0.141	0.150	0.935
**Q16**	0.031	0.013	0.090	**0.910**	0.073	0.123	0.858
**Q17**	0.013	**0.740**	0.150	0.044	-0.046	-0.155	0.599
**Q18**	0.042	**0.944**	-0.033	-0.010	0.020	0.101	0.905
**Q19**	0.055	**0.913**	-0.071	-0.020	0.027	0.076	0.848
**Q20**	0.044	**0.923**	0.051	0.030	0.028	0.085	0.865
**Q21**	0.105	0.122	-0.034	0.203	0.148	**0.857**	0.825
**Q22**	0.088	-0.049	0.012	0.378	0.159	**0.769**	0.769

F1 contained Q1, Q2, Q3, Q4, F2 contained Q17, Q18, Q19 and Q20, F3 contained Q9, Q10, Q11, and Q12, F4 contained Q14, Q15, Q16, F5 contained Q5, Q6, Q7 and Q8, F6 contained Q21, Q22

### Confirmatory factor analysis

**Fig. 1:**
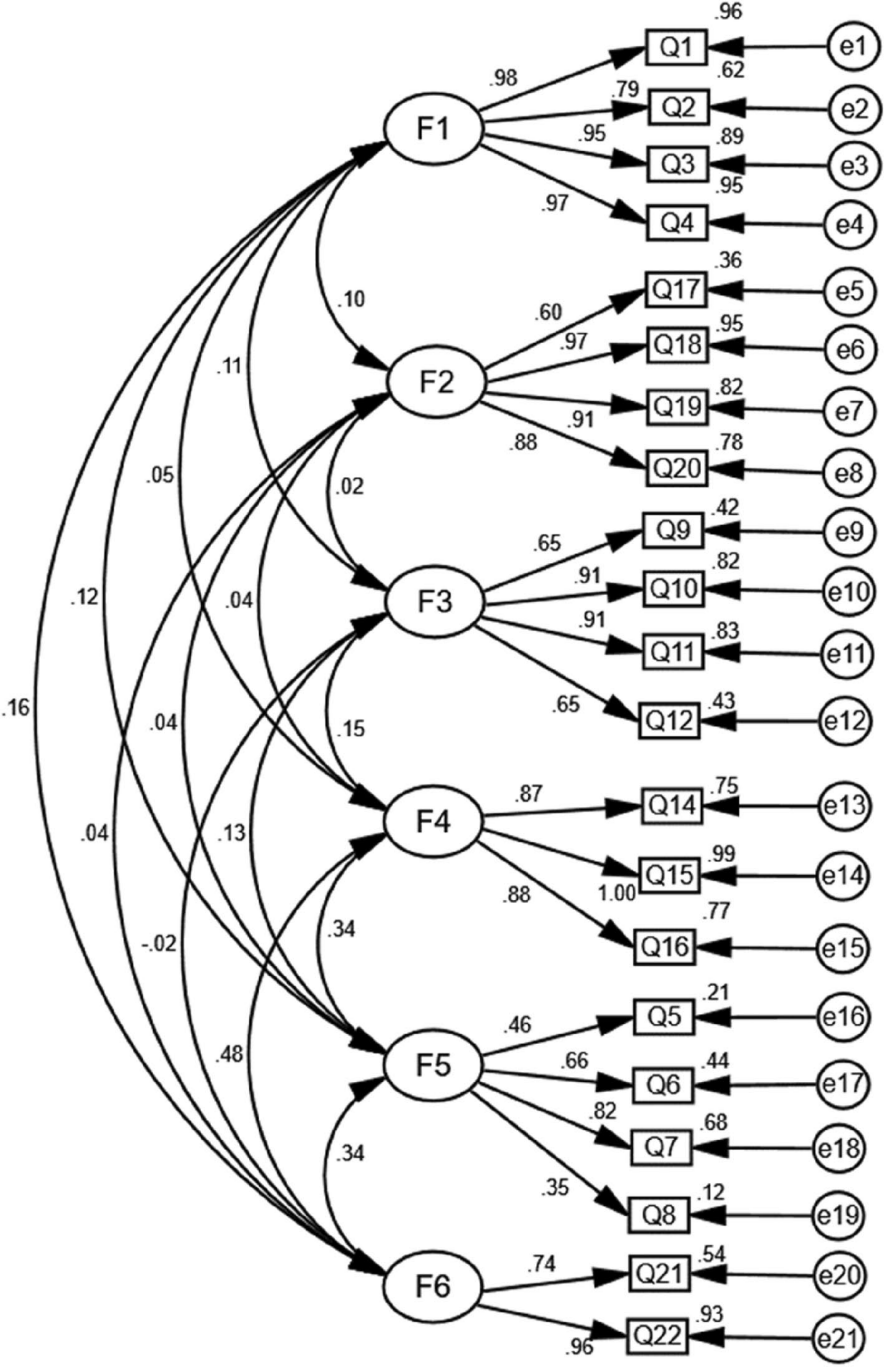
Standardized six-factor structural model of the Chinese version of the Hypertension Belief Assessment Tool Scale (Initial model). F1 (Perceived susceptibility to hypertension, four items); F2 (Perceived barriers to taking action, four items); F3 (Perceived socio-economic-related severity, four items); F4 (Perceived benefits of taking action, three items); F5 (Perceived severity of hypertension, four items) and F6 (Self-efficacy, two items)

**Fig. 2:**
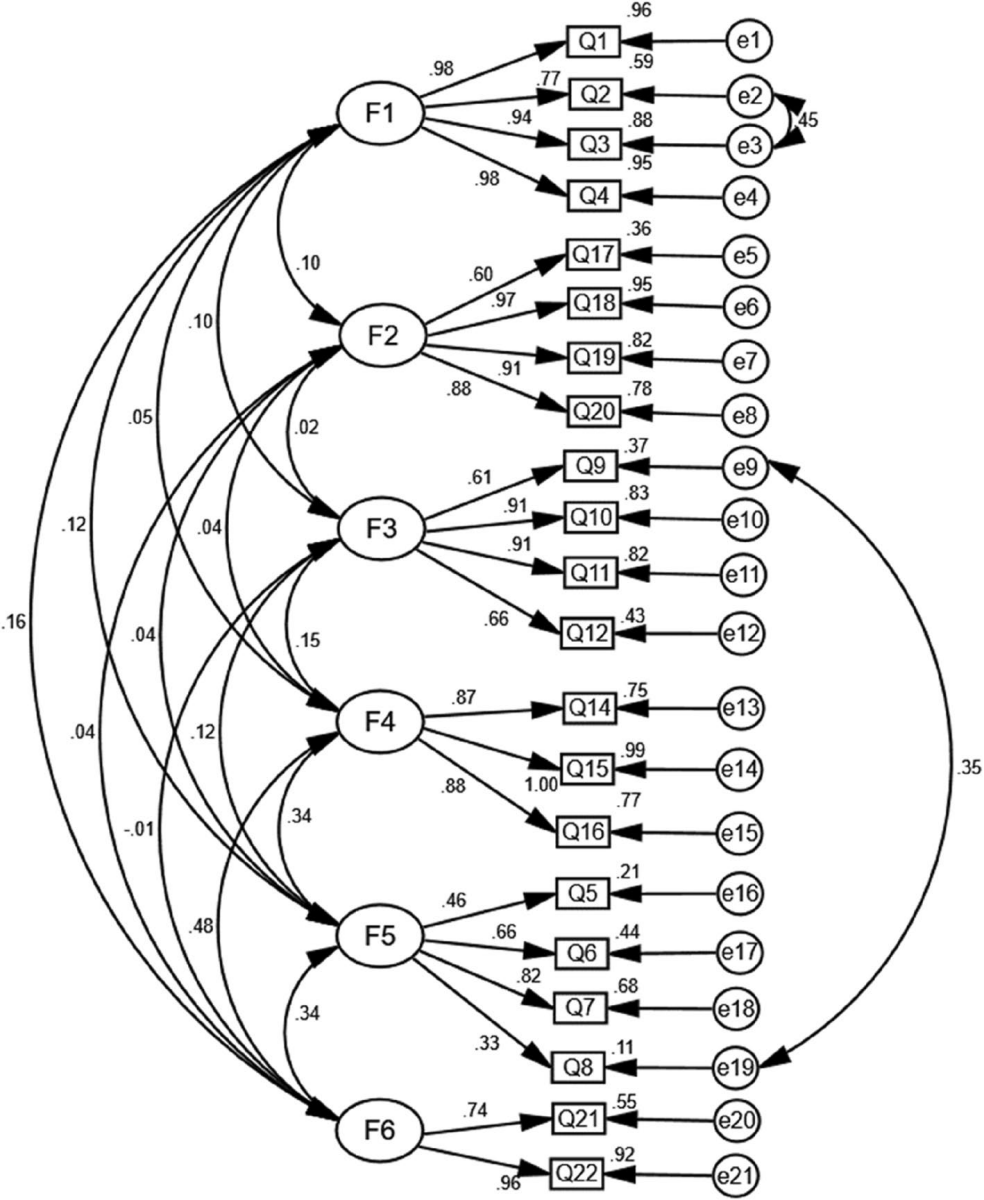
Standardized six-factor structural model of the modified Chinese version of the Hypertension Belief Assessment Tool Scale (Final model). F1 (Perceived susceptibility to hypertension, four items); F2 (Perceived barriers to taking action, four items); F3 (Perceived socio-economic-related severity, four items); F4 (Perceived benefits of taking action, three items); F5 (Perceived severity of hypertension, four items) and F6 (Self-efficacy, two items)

Goodness-of-fit is evaluated using a range of model fit indices, which assess the relationship between the theoretical information and the observed information [48]. With CFA, the initial fit indices were not perfect (Fig. 1). Then we adjusted the modification indices to improve the fit indices [49]. Six-factor model existed and manifested an acceptable index, with CMIN/DF = 2.491, CFI = 0.952, IFI = 0.952, TLI = 0.941, and RMSEA = 0.068 (Table 7; Fig. 2).

**Table 7:** Evaluation fitness of structural equation model

Model	CMIN/DF	NFI	RFI	IFI	TLI	CFI	PNFI	PCFI	RMSEA
Initial model	3.027	0.905	0.885	0.934	0.920	0.934	0.750	0.774	0.071
Modified model	2.491	0.923	0.905	0.952	0.941	0.952	0.756	0.780	0.068
Standard value	< 5.000	> 0.900	> 0.900	> 0.900	> 0.900	> 0.900	> 0.500	> 0.500	< 0.080

### Discriminant validity

In our sample, the total scores on the HBAT were ranked from high to low in SPSS. The first 27% of the total was classified as the high subgroup and the last 27% of the total as the low subgroup, with the low subgroup coded as 1 and the high subgroup coded as 2 [50]. Subsequently, we performed an independent samples t-test for high and low groups. Table 8 showed that there was a significant difference between the two groups (*P* < 0.001), which indicated the discriminant validity was acceptable.

**Table 8:** Discriminant validity analysis in HBAT of 21 items (*N* = 325)

Items	Low-score group Mean ± SD	High-score group Mean ± SD	* t*	* P*
**Q1**	1.65 ± 1.02	3.77 ± 0.85	-15.215	< 0.001
**Q2**	1.50 ± 0.72	3.25 ± 0.91	-14.398	< 0.001
**Q3**	1.58 ± 0.85	3.67 ± 0.88	-16.174	< 0.001
**Q4**	1.57 ± 0.91	3.88 ± 0.93	-16.901	< 0.001
**Q5**	3.61 ± 0.85	4.86 ± 0.34	-8.475	< 0.001
**Q6**	4.55 ± 0.79	3.10 ± 0.76	-3.360	0.001
**Q7**	4.48 ± 0.67	4.87 ± 0.42	-4.603	< 0.001
**Q8**	3.56 ± 0.87	4.10 ± 0.82	-4.313	< 0.001
**Q9**	2.41 ± 0.98	3.10 ± 0.69	-5.333	< 0.001
**Q10**	2.04 ± 0.70	2.81 ± 0.67	-7.653	< 0.001
**Q11**	2.03 ± 0.72	2.82 ± 0.66	-7.759	< 0.001
**Q12**	2.13 ± 0.67	2.79 ± 0.68	-6.650	< 0.001
**Q14**	4.56 ± 0.63	4.96 ± 0.22	-5.639	< 0.001
**Q15**	4.62 ± 0.51	4.98 ± 0.10	-6.699	< 0.001
**Q16**	4.58 ± 0.57	4.95 ± 0.25	-5.565	< 0.001
**Q17**	2.13 ± 1.01	2.96 ± 0.75	-6.294	< 0.001
**Q18**	1.87 ± 0.71	2.86 ± 0.63	-9.861	< 0.001
**Q19**	1.91 ± 0.77	2.97 ± 0.69	-9.547	< 0.001
**Q20**	1.82 ± 0.71	2.84 ± 0.57	-10.651	< 0.001
**Q21**	4.04 ± 1.03	4.79 ± 0.47	-6.282	< 0.001
**Q22**	4.28 ± 0.62	4.79 ± 0.47	-6.206	< 0.001

### Internal consistency reliability

The instrument was found to have a Cronbach’s α value of 0.803. The reliability of the dimension of susceptibility was 0.958, the dimension of severity was 0.629, the dimension of socio-economic-related was 0.851, the dimension of benefits was 0.933, the dimension of barriers was 0.901, and the dimension of self-efficacy was 0.802. In Item-total score correlations, there was a strong correlation and statistical significance, and the correlations ranged from 0.260 to 0.677, which suggested that the items all belonged to the scale [40] (Table 9). Overall, there are 21 items in the HBAT, and most of the factors are the same as in the original model.

**Table 9:** Item-total score Pearson correlation analysis results in HBAT of 21 items (*N* = 325, α = 0.05)

Items	Item content	* r*	* P*
**Q1**	My chance of developing hypertension is high	0.644	< 0.001
**Q2**	The possibility that I will develop hypertension in a few years is very high	0.610	< 0.001
**Q3**	I can have hypertension even without the feeling of its symptoms	0.674	< 0.001
**Q4**	I am more likely to catch HTN than other people	0.677	< 0.001
**Q5**	Hypertension is a serious disease	0.462	< 0.001
**Q6**	Hypertension is a lifelong disease	0.260	< 0.001
**Q7**	Complication of hypertension can cause permanent damage	0.349	< 0.001
**Q8**	Hypertension can cause death	0.290	< 0.001
**Q9**	Sudden falling may happen due to hypertension	0.325	< 0.001
**Q10**	Hypertension will cause dependence on others	0.433	< 0.001
**Q11**	Hypertension can threaten patients’ relationship with their family	0.441	< 0.001
**Q12**	Hypertension can cause sexual dysfunction	0.426	< 0.001
**Q14**	Early detection of HTN makes prevention of complications easier	0.376	< 0.001
**Q15**	Timely initiation of treatment makes prevention of complications easier	0.410	< 0.001
**Q16**	Keeping blood pressure close to normal prevents hypertension complications	0.375	< 0.001
**Q17**	Lack of health information can prevent getting healthcare services	0.348	< 0.001
**Q18**	Lack of transportation makes it difficult for me to get healthcare service	0.437	< 0.001
**Q19**	The cost of screening service makes it difficult for me to get the service	0.418	< 0.001
**Q20**	Lack of nearby health facilities makes it difficult for me to get services	0.469	< 0.001
**Q21**	I can know my status by checking my BP regularly	0.398	< 0.001
**Q22**	I am sure of when to contact health workers while I am feeling unusual health conditions	0.388	< 0.001

### Retest-reliability

For the retest reliability, we evaluated the same group of subjects twice after an interval of two weeks using the same scale and then calculated the correlation coefficient of the two evaluation results. The retest reliability was 0.710. The obtained retest reliability value is greater than 0.7, which is acceptable [51].

## Discussion

The scale has been validated among hypertensive patients in China. The average score was 77.88 ± 8.72. This indicated that the hypertensive patients in this study have moderate health beliefs. There is growing recognition that positive health beliefs can help lower blood pressure and prevent complications. The HBM deems that messages will achieve optimal behavior change if they successfully target perceived barriers and threats [33, 52]. This study was the first to discuss patients’ hypertension beliefs in China. The outcome provided evidence that the Chinese version of the HBAT has good psychometric properties. Therefore, the scale will be a practical tool to investigate the beliefs about Hypertension in Chinese hypertensive patients. A total of 2 items was removed from this study, and 21 items were finally retained. The 21-item tool contributes to filling the gap in measures of hypertension beliefs in hypertensive patients. Positive health beliefs are associated with lower blood pressure [20]. The HBAT can help hypertensive patients effectively measure their sense of self-belief, and at the same time, through the assessment of the scale, it is beneficial for patients to enhance their sense of belief in the treatment of the disease and to improve their health awareness and health level. It also helps healthcare workers understand the management of Hypertension. Scale screening can help healthcare workers identify and intervene in the disease process at an early stage and promote public health. This study demonstrated that the HBAT scale has good psychometric properties, which will be an important reference for the psychological assessment of elderly hypertensive patients. It deserves to be promoted and applied in hypertensive populations.

### The validity of the scale is acceptable

The content validity and construct validity of the scale were examined in our sample. The results of content validity are acceptable. Construct validity refers to the theoretical structure and characteristics measured by a test [53]. In the Treatment Adherence Questionnaire for patients with Hypertension, the EFA supported six factors with a total variance explained of 54%, and Cronbach’s alpha for the total scale was 0.74 [54]. Compared to these scales, this study demonstrated a better explanation of variance.

In our sample, the 21 items were divided into corresponding dimension ranges, and the distribution of most items was consistent with the original scale, except for Q9 (“Sudden falling may happen due to hypertension”). Q9 belonged to the dimension of perceived socio-economic-related severity due to its high factor loading of 0.749 in factor 3 in our study. The reasonable explanation was that all people supposed falling could not only lead to dependence on others but also cause many unnecessary troubles in the socio-economic aspects. This may have something to do with differences between cultures and people’s ability to understand. In the factor analysis, we found that the factor loading of Q13 was less than 0.50 on any dimension. Finally, we removed Q13 from the scale. In the original scale, KMO in the initial solution was 0.84 (*P* < 0.01), and the six extracted factors accounted for 70.061% of the total variance [36]. Compared to the original scale, the 21 HBAT could explain 77.898% of the variance, which indicates a suitable explanation of the variance.

Results from the two-tailed independent samples t-test also illustrated that the top score group and low score group showed a significant difference (*P* < 0.001) in our survey. Therefore, it describes excellent discriminant validity.

### The scale has excellent reliability

In Item-total score correlation analysis, Q23 has the lowest correlation coefficient with the total score of the scale (*r* = 0.252). Therefore, Q23 was rejected in the latter analysis. As predicted by the literature, the Cronbach’s alpha of more than 0.70 is recommended [55]. The HBAT indicates good homogeneity, and the overall Cronbach’s alpha was 0.803 in this study. In comparison with the 18-item Facilitators of and Barriers to Adherence to Hypertension Treatment Scale (Four factors, the Cronbach’s alpha was 0.78) [56]. The 21-item HBAT scale has six factors, a satisfactory Cronbach’s alpha, with appropriate reliability and validity. The use of this measurement tool for Chinese hypertensive patients is feasible and shows reliable consequence.

### The scale has good applicability

The results demonstrated that the HBAT has a stable construct and discriminant validity. Further, it is of great importance to explore the beliefs of hypertensive patients. We reckon that older participants, with better knowledge of Hypertension, high financial income, and those who have a chance to act would perceive more severity of Hypertension and more susceptible to Hypertension, and understand the benefits of healthy belief better [57]. The HBM is one of the most popular and widely utilized theories in public health [52]. Therefore, investigating the beliefs of Hypertension will be beneficial to the management and control of chronic diseases in the future. A health belief model-based instrument was also found to be an appropriate instrument for measuring health beliefs for the prevention of osteoporosis [58]. According to the literature, the implementation of a knowledge-attitude-belief-practice model for patients was beneficial to improve disease awareness, medication compliance, self-efficacy, healthy behavior, and quality of life [59]. Hence, medical care personnel should use the HBAT (Chinese version) to obtain the health beliefs of patients with Hypertension, and then provide health education and medical support to patients.

## Conclusion

The study investigates the psychometric properties of the HBAT (Chinese version) in Chinese hypertensive patients. It is a reliable and valid instrument for assessing the beliefs of Chinese hypertensive patients.

### Limitations

Our study sample size was limited. The present study was conducted only in one hospital in Anhui Province. Therefore, future research should expand the sample to verify the result better.

## Data Availability

The datasets used and/or analyzed during the current study available from the corresponding author on reasonable request.

## Abbreviations

HBAT: Hypertension Belief Assessment Tool
EFA: Exploratory Factor Analysis
CFA: Confirmatory Factor Analysis
CMIN/DF: Chi-square/Degree of Freedom
CFI: Comparative Fit Index
IFI: Incremental Fit Index
RMSEA: Root-mean-square Error of Approximation
TLI: Tucker Lewis Index
HBM: Health Belief Model
KMO: Kaiser–Meyer–Olkin
